# Predicted impacts of climate warming on aerobic performance and upper thermal tolerance of six tropical freshwater fishes spanning three continents

**DOI:** 10.1093/conphys/coy056

**Published:** 2018-10-15

**Authors:** Dominique Lapointe, Michael S Cooperman, Lauren J Chapman, Timothy D Clark, Adalberto L Val, Marcio S Ferreira, John S Balirwa, Dismas Mbabazi, Matthew Mwanja, Limhong Chhom, Lee Hannah, Les Kaufman, Anthony P Farrell, Steven J Cooke

**Affiliations:** 1 St. Lawrence River Institute of Environmental Sciences, Cornwall, ON, Canada; 2 Fish Ecology and Conservation Physiology Laboratory, Department of Biology and Institute of Environmental Science, Carleton University, Ottawa, ON, Canada; 3 Gordon and Betty Moore Center for Science, Conservation International, Arlington, VA, USA; 4 Department of Biology, McGill University, Montreal, QC, Canada; 5 Deakin University, School of Life and Environmental Sciences, Geelong, Victoria, Australia; 6 Brazilian Institute for Research of the Amazon—INPA, Manaus, AM, Brazil; 7 National Fisheries Resources Research Institute—NaFIRRI, Jinja, Uganda; 8 Aquaculture Research and Development Center—ARDC, NaFIRRI (Kajjansi), Kampala, Uganda; 9 University of Battambang, Battambang, Cambodia; 10 Bren School of Environmental Science & Management, University of California, Santa Barbara, CA, USA; 11 Department of Biology, Boston University, Boston, MA, USA; 12 Zoology Department and Faculty of Land and Food Systems, University of British Columbia, Vancouver, BC, Canada

**Keywords:** Aerobic scope, climate change, critical thermal maximum, food security, tropical inland fisheries

## Abstract

Equatorial fishes, and the critically important fisheries based on them, are thought to be at-risk from climate warming because the fishes have evolved in a relatively aseasonal environment and possess narrow thermal tolerance windows that are close to upper thermal limits. We assessed survival, growth, aerobic performance and critical thermal maxima (CTmax) following acute and 21 d exposures to temperatures up to 4°C higher than current maxima for six species of freshwater fishes indigenous to tropical countries and of importance for human consumption. All six species showed 1.3–1.7°C increases in CTmax with a 4°C rise in acclimation temperature, values which match up well with fishes from other climatic regions, and five species had survival >87% at all temperatures over the treatment period. Specific growth rates varied among and within each species in response to temperature treatments. For all species, the response of resting metabolic rate (RMR) was consistently more dynamic than for maximum metabolic rate, but in general both acute temperature exposure and thermal acclimation had only modest effects on aerobic scope (AS). However, RMR increased after warm acclimation in 5 of 6 species, suggesting incomplete metabolic compensation. Taken in total, our results show that each species had some ability to perform at temperatures up to 4°C above current maxima, yet also displayed certain areas of concern for their long-term welfare. We therefore suggest caution against the overly broad generalization that all tropical freshwater fish species will face severe challenges from warming temperatures in the coming decades and that future vulnerability assessments should integrate multiple performance metrics as opposed to relying on a single response metric. Given the societal significance of inland fisheries in many parts of the tropics, our results clearly demonstrate the need for more species-specific studies of adaptive capacity to climate change-related challenges.

## Introduction

Inland fisheries can be critical to the food security of developing nations in tropical regions (Béné *et al.*, 2015; Youn *et al.*, 2014). For example, freshwater fish can account for 60–75% of the total dietary protein intake in countries of tropical Africa, Asia and the neo-tropics (FAO, 2014; Hortle, 2007), and consumption of fish with high contents of fatty acids, amino acids, vitamins and minerals can offset micronutrient deficiencies (Kawarazuka and Béné, 2011; Tacon and Metian, 2013). Inland fisheries can also be central to national and regional poverty alleviation strategies of many tropical countries by providing a primary source of income via sale of fish, a means for barter, stable full time and seasonal employment, and commercial development opportunities with associated taxable revenue (Béné *et al.*, 2009; Neiland and Béné, 2006; Nunan, 2013). Indeed, Allison *et al.* (2009) estimated that inland fisheries-related activities (capture fisheries, aquaculture, fish processing and fish trade) provide income for approximately 10% of the global population, including many people that are among the poorest and most disenfranchised on the planet. Furthermore, inland fisheries of the tropics typically occur in locations of high taxonomic diversity and involve indiscriminate harvest of all available species and sizes (Kolding and van Zwieten, 2014; McIntyre *et al.*, 2016; Welcomme *et al.*, 2010). As such, the status and conduct of tropical inland fisheries is directly associated with efforts to protect global biological diversity.

Given the significance of tropical inland fisheries to both human well-being and biodiversity conservation, it is important to understand how fish populations that underlie these fisheries will respond to environmental change including climate warming. As with other ectothermic animals, water temperature controls and limits a wide range of physiological processes and activities in fishes (Fry, 1947, 1971). Tropical species, in general, have evolved in warm and relatively thermo-stable environments and, as such, are predicted to (a) have narrower thermal windows than their temperate counterparts, (b) be living near the upper edge of their thermal tolerance limit and (c) lack adaptive capacity to rising temperatures as they have never experienced these conditions in their recent evolutionary past (Janzen, 1967; Stillman, 2003; Tewksbury *et al.*, 2008). Hence, tropical ectotherms are presumed to be especially sensitive to the temperature increases predicted with climate change. This suite of characteristics describing the presumed sensitivity of tropical species to warming was initially captured by Rapoport’s Rule (Stevens, 1989) and has come to be termed the ‘climatic variability hypothesis’ (CVH) (Addo-Bediako *et al.*, 2000).

However, a clear lack of empirical data on the thermal optima, tolerances, acclimation capacities and potential for adaptive heritable change of tropical freshwater fishes makes it challenging to evaluate the true magnitude of threat of rising temperatures and the applicability of the CVH to tropical fish and fisheries (Ficke *et al.*, 2007). For fishes and other aquatic ectotherms, aerobic scope (AS), which is the temperature-dependant difference between the standard metabolic rate (SMR; the minimum aerobic metabolic rate needed to support life) and the maximum metabolic rate (MMR; the maximum rate of aerobic metabolism), is a performance trait that has been hypothesized to govern thermal tolerance and fitness (Pörtner, 2002; Pörtner *et al.*, 2017), because all activities require oxygen either immediately or as a repayment of an oxygen debt (Farrell, 2016). Thus, a large AS theoretically provides a greater capacity to simultaneously power multiple performance-related activities such as growth, feeding, predator avoidance and reproduction (Farrell, 2016; Fry, 1947). This hypothetical framework, coined oxygen- and capacity-limited thermal tolerance (OCLTT; Pörtner, 2010), has provided a potentially useful conceptual structure for predicting how ectotherms may respond to thermal stress (Farrell *et al.*, 2009, 2008; Pörtner and Farrell, 2008; Pörtner and Peck, 2010). Even so, empirical studies have challenged the broader applicability of the OCLTT hypothesis because many results are in conflict with predictions (Clark *et al.*, 2011, 2013; Ern *et al.*, 2016; Gräns *et al.*, 2014; Norin and Clark, 2016; Poletto *et al.*, 2017; Raby *et al.*, 2016). Also, acute thermal tolerance has proved to be independent of AS (Brijs *et al.*, 2015; Ern *et al.*, 2016).

To evaluate the applicability and utility of the CVH and OCLTT hypotheses with regard to predicting the thermal performance of data-poor inland tropical fishes, we conducted a broad-scale series of thermal challenge experiments using six species across three continents. Specifically, we conducted acute (16 h) exposure and 21 d thermal acclimation experiments involving measures of AS and other proxies of temperature-specific performance on two species of fish important as human food from each of the three equatorial study countries (Brazil, Uganda and Cambodia). Evidence in support of the CVH would be all six of the tested species showing notable levels of deleterious effects from elevated water temperatures. Evidence against the broad applicability of the CVH to tropical freshwater fishes would be either no response to thermal challenge or a range of different species-specific responses. Our evaluation of the OCLTT hypothesis involves comparing temperature-specific AS to other measures of thermal performance such as survival, growth rates and body condition (measured here as weight-at-length). Support of OCLTT applicability to tropical freshwater fishes would be a close match between the thermal performance curve of AS and the thermal performance curves of other measures of temperature-specific performance (i.e. survival, growth and weight-at-length), including a match in optimal temperatures of each of the measured variables.

## Materials and Methods

### Fish supply and holding

**Figure 1:**
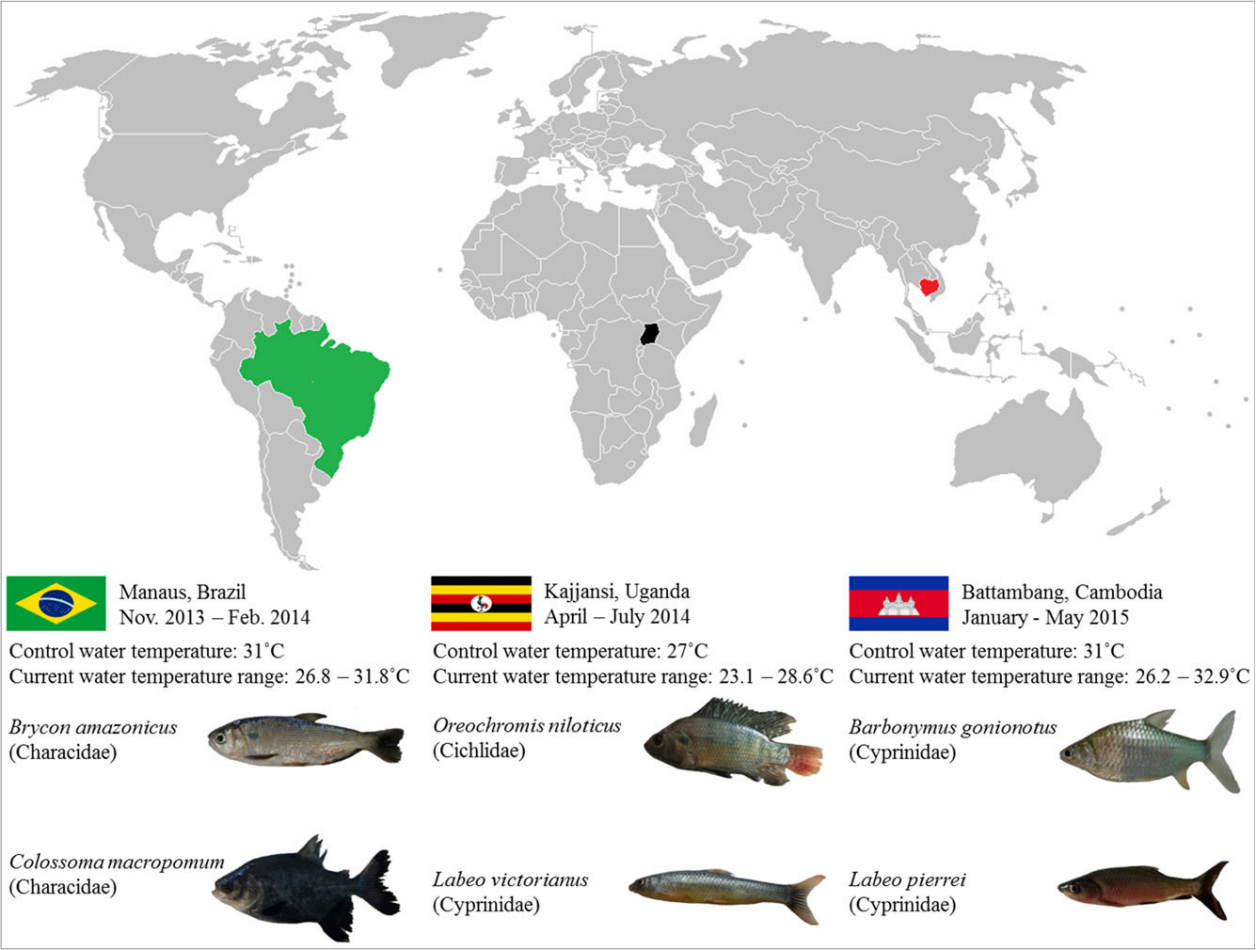
Study locations, site-specific information on water temperature and selected species. The current water temperature range for Brazil was determined using water temperature data for the Rio Negro provided by INPA (personal communication). In Uganda, water temperature data for Lake Victoria and the Sio River were provided by the National Fisheries Resources Research Institute and used to determine the range. Lastly, the current water temperature range reported for Cambodia was based on data for the Tonle Sap Lake provided by Cambodia’s Inland Fisheries Research and Development Institute. The control temperature for thermal acclimation was defined as the average maximum water temperature observed at each location, whereas current water temperature range corresponds to the range of ambient water temperature encountered in the natural habitat of the species.

We selected fish species of known importance as local human food, which are prevalent in regional fisheries and aquaculture, and are native to that region (Fig. 1). In Brazil, we purchased Age-0 Matrinxã (*Brycon amazonicus*; Spix & Agassiz 1829) (mean initial mass ± SEM 7.1 ± 0.2 g), and Tambaqui (*Colossoma macropomum*; Cuvier 1816) (mean initial mass ± SEM 27.5 ± 0.7 g) from Aquaculture Santo Antonio (Manaus, AM, Brazil). *Brycon amazonicus* are benthopelagic, found throughout the primary rivers and floodplains of western Amazonia basin and undertake complex and long distance longitudinal and lateral migrations. *Colossoma macropomum* are found throughout the Amazon and Orinoco basins, where they undertake seasonal lateral migrations from the river channel onto inundated floodplains (Val and Almeida-Val, 1995). Both species are important in the dispersal of seeds from the plants of the flooded forest. At the aquaculture facility, fingerlings had been hatched and reared in tanks supplied with surface waters from the Amazon River and open to natural light cycles. The brood line for these fingerlings was initiated less than 10 years earlier and is continuously infused with wild captured adults, so we assume domestication effects were minimized (*pers. comm*., manager, Aquaculture Santo Antonio). Water temperature in the rearing tanks ranged between 29 and 33°C, which is similar to the lake environments and margins of Amazonian rivers where both species are found (Araujo-Lima and Goulding, 1998; Lowe-McConnell, 1987). Fingerlings were transported to our laboratory at the Brazilian Institute for Research of the Amazon (INPA, Manaus, AM, Brazil) in November 2013.

In Uganda, we purchased from local fishers Age-0 wild Nile tilapia (*Oreochromis niloticus*; Linnaeus 1758) (mean initial mass ± SEM 26.8 ± 0.6 g) and Ningu (*Labeo victorianus*; Boulenger 1901) (mean initial mass ± SEM 24.9 ± 1.2 g) captured in Lake Victoria in April and June 2014, respectively. Native members of the trans-Sudanian fish fauna, *O. niloticus* were introduced and naturalized to Lake Victoria in the 1950s (Reinthal and Kling, 1994), while *L. victoranus* are native to the Lake Victoria basin. *Oreochromis niloticus* is commonly found in the shallow waters of lakes and slow moving river sections of tropical and sub-tropical Africa and does not undertake largescale movements. *Labeo victorianus* populations of the lakes of eastern tropical Africa were potamadromous historically, but habitat loss and related pressures appear to be forcing a shift away from migration and to more restricted life history movements. Both species are omnivorous (Riede, 2004). On the day of collection, the captured fish were transported to the Aquaculture Research and Development Center of the National Fisheries Resources Research Institute (Kajjansi, Uganda).

In Cambodia, we purchased Age-0 Java barb (*Barbonymus gonionotus*; Bleeker 1850) (mean initial mass ± SEM 27.9 ± 0.5 g) from a family-owned aquaculture company in Bak Am Rek (Battambang Province, Cambodia) in February 2015, and Age-0 Trey pawa (*Labeo pierrei*; (Sauvage 1880) (mean initial mass ± SEM 20.4 ± 0.3 g)) from Sourm Pam Farm (Otaki, Battambang Province, Cambodia) in March 2015. *Barbonymus gonionotus* are primarily benthopelagic in lakes and slow moving river segments of the Mekong River drainage. Their diet is primarily vegetation and they undertake seasonal migrations onto inundated floodplains (P. Channg, IFReDI, *pers. comm*.). *Labeo pierrei* are migratory species primarily found in the larger rivers of the Mekong basin where they graze on algae (P. Channg, IFReDI, *pers. comm*.). For both species, individuals had been captured as fry in the wild and brought to the aquaculture facility for grow-out. Specimens were transported to our laboratory at the University of Battambang.

At all locations, upon arrival to the laboratory fish were held in outdoor tanks provisioned with natural light regimes and flow-through water. In Brazil, we used groundwater at a constant 28°C, while in Uganda and Cambodia water supply was via above ground storage tanks with temperatures ranging between 23–28°C and 25–31°C, respectively. At all locations, fish were fed once daily *ad libitum* with commercially prepared fish pellets during the outdoor holding period, and fish were held at the laboratory a minimum of 14 d prior to the start of temperature manipulations. Because logistical constraints dictated that we executed our experiments with one species at a time at each location, the second species remained in the holding tanks under conditions described above for approximately 44 d before usage. All animal care and experimental procedures were approved by the Animal Care Committee of Carleton University (Protocol #100 481), and complied with the guidelines of the Canadian Council on Animal Care. In addition, our work in Brazil complied with INPA’s protocol 047/2012 of the Ethics Committee on Animal Experiments. Fish capture and/or transport permits were obtained from local authorities.

### Thermal acclimation

At commencement of experiments with each species, individual fish of a species were randomly selected from the holding tank, anesthetized using clove oil (40 ppm), weighed, measured and given a unique visible implant elastomer tag (VIE; Northwest Marine Technology, Inc., Shaw Island, WA, USA). A combination of three colours (red, yellow and orange) and five tagging sites (caudal, dorsal, pectoral, pelvic and anal fins) provided 15 unique IDs. Upon tagging, fish were randomly distributed into the 9-treatment tanks that were part of the treatment system (similar design at each location). Each of the 9-treatment tanks held 15 uniquely tagged individuals, and all tanks were set at the same temperature as the holding tank. The temperature treatment system consisted of three independent re-circulating treatment loops, with each loop consisting of a 310-l head tank and three 150 l fish treatment tanks (replicates). Each loop was equipped with bio-physical canister filters with integrated water pumps, aeration pumps and air stones, a UV filter, and a thermostat controlled 1000 W heating element. Filter pumps circulated water within each loop at a rate of 175 l h^−1^ and new water trickled into the head tank at a rate of 25 l h^−1^ with overflow discharged to a drain. Additionally, ~30 fish were placed into the head tank of the ‘control’ temperature treatment line for use in acute exposure experiments (described below). Throughout the course of the experiments, water temperature and dissolved oxygen concentration in all tanks were measured twice daily using a hand-held metre (Handy Polaris, OxyGuard, Farum, Denmark).

Following 2 days of post-tagging recovery, we increased water temperature in the treatment loops at a rate of 1°C d^−1^ until target temperatures of control, control +2°C and control +4°C were reached. Note that we defined ‘control’ temperature for each species as the current average maximum temperature encountered in the natural habitat of the species (i.e. average water temperature at the warmest time of the day during the warmest month of the year), as determined from the best water quality data available for a location. Thus, the control temperature did not necessarily match the prevailing ambient temperature at the time of fish acquisition. Control water temperatures in Brazil, Uganda, and Cambodia were 31°C, 27°C and 31°C, respectively (Fig. 1). The treatment temperatures were control +2°C and control +4°C, and were intended to represent realistic projections from global warming models (www.climatewizzard.org) of water temperature increase that can be expected in a 20–40 year timeframe based on ‘average’ carbon emission scenarios. In the temperature treatment tanks, fish were fed *ad libitum* once daily with known quantities of commercially prepared food every 10 min until some remained at the surface. Each tank was cleaned 30 min after the last ration and all leftover food particles were collected to minimize the impact of feeding on water quality. Subsets of fish from the control treatment in the head tank were used in acute exposure experiments described below, while all other individuals were held in the treatment tanks for a 21-d thermal acclimation period. During thermal acclimation, tanks were visually examined twice daily for fish showing signs of injuries or disorientation and moribund or deceased fish were promptly removed from the tanks.

### Respirometry systems

We used an intermittent-flow respirometry system (Norin and Clark, 2016; Schurmann and Steffensen, 1997) following best practices described in Clark *et al.* (2013) to measure the oxygen uptake rates $\left(M_{O_{2}}\right)$ of individual fish (mg O_2_ kg^−1^ min^−1^) from which we then derived resting metabolic rate (RMR) and maximum metabolic rate (MMR). We used eight rectangular 2 l polypropylene respirometers simultaneously (one fish per respirometer), with respirometers submerged in a shared water bath containing recirculated, filtered, UV sterilized and aerated water. Water was continuously circulated through each respirometer via a recirculation loop of plastic tubing equipped with an in-line water pump (model 1046, EHEIM GmbH & Co., Deizisau, Germany). In cases where an individual fish was below a threshold size, we placed an inert acrylic block into its respirometer to reduce water volume inside the chamber. We used FireSting fibre-optic oxygen sensors and contactless oxygen sensor spots (PyroScience GmbH, Aachen, Germany) positioned within the recirculation loop of each respirometer to record oxygen levels at 0.5 Hz. Water in the respirometers was renewed from the surrounding water bath using automated flush pumps equipped with continuous cycle-timers, whereby flushing occurred for 10 min followed by a 5-min period when the respirometer was sealed. Temperature in the water bath was maintained by two 1000 W heaters connected to digital thermostats, and temperature was validated using a hand-held metre. Background respiration in each respirometer was measured at the beginning and end of each respirometry trial. Fish $M_{O_{2}}$ was corrected by assuming a linear change in background respiration between these two measurements. For all respirometry work, feeding of fishes ceased 48 h prior to placement into respirometers to ensure fish were post-absorptive.

### Respirometry protocol

Between 2 and 15 d after stocking of the treatment tanks (the specific timing dependent upon logistic considerations unique to each of the six species), we selected eight individuals from the control treatment head tank for determination of RMR and MMR when exposed to acute changes in water temperature (termed ‘acute’ exposure). Each fish was placed into a respirometry chamber submerged in the water bath maintained at the control temperature and dissolved oxygen levels were measured for at least 16 h (including overnight), resulting in between 64 and 80 individual $M_{O_{2}}$ measurements per fish (10:5 min flush:seal cycles). These data were used to determine RMR as described below. Subsequently, one fish at a time was transferred into a 30-l circular exercise tank and gently prodded to produce short periods of burst swimming for 3 min, followed by 1 min of air exposure during which time each fish was weighed. This exhaustive exercise protocol has been shown to elicit maximum levels of metabolism in fish species that do not naturally swim for prolonged durations (Clark *et al.*, 2013; Norin and Clark, 2016), and was selected for the present study to ensure standardization across species. Following air exposure, the fish was returned to its respirometer and oxygen consumption measurements resumed immediately by sealing the respirometer manually (for 5–10 min) before returning to the 10:5 min flush:seal cycle for at least another 2 h. These data were then used to determine MMR as described below. Upon completion of the MMR trials at the control temperature, fish were kept in respirometers as water temperature in the respirometry system was increased to control +2°C at a rate of 2°C h^−1^. We then repeated the methodology for RMR and MMR following the above procedures (including >16 h of overnight measurements from which to calculate RMR), and repeated it once again after increasing the temperature to control +4°C. Finally, we decreased water temperature back to the control value for a final assessment of RMR and MMR to test for repeatability (again with >16 h of data for RMR calculations). To control for the effect of treatment order on $M_{O_{2}}$, we repeated the entire protocol with a different batch of 8 fish taken from the control temperature head tank using a different sequence of temperatures (control +2°C, control +4°C, control, control +2°C). Fish used in these acute exposure trials were not used in any subsequent experiments.

At 21 d after the start of the temperature treatments, we randomly selected eight fish from a single treatment tank and transferred them into respirometers for determination of RMR and MMR at their respective treatment temperature, following methods described above. We refer to these trials as ‘post-acclimation’ (PA), after which each fish was euthanized via cerebral percussion, weighed (g), and measured (standard, fork and total length, in mm). We repeated the above until eight fish from each of the nine treatment tanks had been tested (tank order within a given temperature was randomly determined).

### Critical thermal maximum

Also at 21 d after the start of the temperature treatments, we followed methods of Fangue *et al.* (2006) and Chen *et al.* (2013) to determine the critical thermal maximum (CTmax), defined as the temperature at which an individual lost equilibrium continuously for 10 s. Working with fish of one replicate at a time, we transferred four randomly selected individuals into a 50-l insulated and aerated tank filled with water at the treatment temperature. Water temperature was controlled using a 1000-W heater connected to a digital thermostat. Fish were left undisturbed overnight. The following day, we increased water temperature at a rate of 0.3°C min^−1^ and continued the increase until each individual had reached their CTmax, upon which the individual was immediately transferred to a recovery tank kept at the initial treatment temperature. We repeated the above for groups of four fish from each of the nine treatment tanks (on a given day, same replicate randomly selected for the post-acclimation trials). Fish used in CTmax trials were not used for any other measurements.

### Calculated variables

We calculated survival (%) over the 21-d acclimation period as:

$$\mathrm{Survival}=100\times \left(\frac{n_{f}}{n_{i}}\right)$$

where *n*_*f*_ and *n*_*i*_ represent the number of individuals in a replicate at the end and at the beginning of the treatment period, respectively. We calculated individual specific growth rate (SGR; % d^−^^1^) as:

$$\mathrm{SGR}=100\times \left[\frac{\ln\;W_{D}-\;\ln\;W_{d}}{D-d}\right]$$

where *W*_*D*_ is final mass (g) determined following euthanasia, *W*_*d*_ is initial mass (g) determined at time of VIE tagging, and *D* − *d* is the time between mass measurements in days (d) (Jobling, 1988).

We determined $M_{O_{2}}$ as the rate of change of O_2_ concentration over time between respirometer flush cycles (∆[O_2_]·t^−1^) using LabChart 7 (ADInstruments Pty Ltd., Bella Vista, NSW, Australia) as:

$$M_{O_{2}}=\left(\Delta \left[O_{2}\right]\;\cdot \;t^{-1}\right)\times V\times W^{-1}$$

where *V* = the respirometer volume (l) and *W* = the mass of the fish (kg). For RMR, we used the *q*_0.1_ method of Chabot *et al.* (2016) and selected the lowest 10% of the $M_{O_{2}}$ values collected during the ~16 h overnight period after eliminating data from the first two hours of the period to avoid elevated values associated with transfer into the respirometer. We also excluded outlier values (±2 standard deviation from the mean; this was never more than four measurements per fish and typically none). We calculated MMR from the highest oxygen consumption rates measured in any 3-min period during the 2 h immediately following the forced exercise and air exposure event. We calculated AS as MMR–RMR and factorial aerobic scope (FAS) as MMR–RMR (Schurmann and Steffensen, 1997). To account for the influence of body mass on rates of oxygen consumption, the acute trial $M_{O_{2}}$ values were standardized to the same species-specific common mass as the fish of the post-acclimation group (*Brycon amazonicus* = 37.38 g; *C. macropomum* = 58.08 g; *O. niloticus* = 28.79 g; *L. victorianus* = 21.27 g; *Barbonymus gonionotus* = 30.90 g; *L. pierrei* = 19.82 g) using the following equation:

$$X_{S}=X_{M}\times {\left[\frac{W_{M}}{W_{F}}\right]}^{SE}$$

where *X*_*S*_ corresponds to the standardized $M_{O_{2}}$, *X*_*M*_ is the measured absolute $M_{O_{2}}$, *W*_*M*_ is the species-specific common fish mass (g), *W*_*F*_ is the final fish mass (g) and SE is the species-specific scaling exponent, because the repeated measures experimental design used in these acute trials prohibited ANCOVA analysis with body mass as the covariate (as was done for the post-acclimation trial data, see below). For a given species, scaling exponents for RMR and MMR correspond to the slope of the bi-logarithmic relationship between $M_{O_{2}}$ and body mass. For RMR, scaling exponents were 0.776, 0.589, 0.918, 0.795, 0.786 and 1.155 for *Brycon amazonicus*, *C. macropomum*, *O. niloticus*, *L. victorianus*, *Barbonymus gonionotus* and *L. pierrei*, respectively. For MMR, scaling exponents were 0.897, 0.735, 0.938, 0.747, 0.800 and 0.825 for *Brycon amazonicus*, *C. macropomum*, *O. niloticus*, *L. victorianus*, *Barbonymus gonionotus* and *L. pierrei*, respectively. These scaling exponents are consistent with the range (0.4–1.29; 0.791±0.011; *n* = 138) compiled for teleost fishes (Clarke and Johnston, 1999). The species-specific common fish mass was subsequently used so that the standardized $M_{O_{2}}$ could be expressed with mass-specific units.

### Statistical analysis

Data from each species were analysed independently. We used SPSS (version 22) for all statistical tests and assessed statistical significance at the 0.05 level. Normality and homogeneity of variance assumptions were verified by examining residuals and by using Levene’s Median test, respectively.

A one-way ANOVA tested for temperature effects on survival during the treatment period. An ANOVA with ‘replicates’ nested within treatment as a factor to control for any potential treatment tank effect tested the influence of temperature on SGR during the treatment period. Differences in fish body size between treatments following the 21-d acclimation were examined using an ANCOVA of log_10_ fish weight by treatment with log_10_ fish standard length as the covariate (i.e. weight-at-length), with ‘replicates’ nested as above. When the interaction between temperature treatment and log_10_ fish standard length was not significant, it was removed from the model. A one-way ANOVA tested treatment temperature effects on CTmax, blocking for replicates as above. When ANOVAs or ANCOVAs revealed a significant effect of temperature treatment on a variable, a Holm–Sidak post hoc test was used to perform pairwise comparisons of the treatments.

Our experimental design provided for two major sets of analyses of the respirometry data (i.e. RMR, MMR, AS, FAS): (i) the response to acute temperature increases describes the response of the fish to rapid temperature change; (ii) the response to a 21-d acclimation to elevated temperatures explores how exposure to elevated temperature affects metabolic rates. Within each species, we also qualitatively compared the acute exposure and post-acclimation results to explore the effects of exposure time on metabolic rates and AS.

*Acute Exposure*—A paired-sample *t*-test measured the repeatability of the first and last RMR and MMR data from the acute exposure trials (i.e. MR measured at the same temperature before and after subjecting specimens to multiple temperatures). Because these data were obtained following two different temperature sequences (increasing vs. decreasing), these paired comparisons were performed for each group separately. If the first and last metabolic parameters (RMR and MMR) did not differ, the mean of the two trials was used. If they differed (at alpha = 0.05), the group was excluded from the subsequent statistical analysis. This conservative approach resulted in eliminating a group for *O. niloticus*, *L. victorianus* and *Barbonymus gonionotus*, whereas *L. pierrei* was entirely omitted. We examined the effect of temperature on MR using a one-way repeated measures ANOVA that included temperature as the within-subject factor. For the two species in Brazil, temperature sequence group was included as a between-subject factor to control for the effect of treatment order on the responses and their interaction.

*Twenty-one day Acclimation*—An ANCOVA with log_10_ fish mass as a covariate assessed temperature treatment effects on log_10_ total RMR, log_10_ total MMR, log_10_ total AS and FAS. Each ANCOVA included temperature as the fixed factor, MR, AS or FAS as the dependent variable, log_10_ fish mass as the covariate and ‘replicates’ nested within treatment. In cases where the interaction between treatment and log_10_ fish mass was not significant, the interaction term was removed and the model run again. A Holm–Sidak post hoc test made pairwise comparisons of the treatment groups. Mass-adjusted estimated marginal means (dependent variable adjusted to a common body mass from the ANCOVA model) allowed reporting of mass-specific units. Acute and post-acclimation data are presented together on figures to permit a qualitative comparison between the different exposure times.

## Results

Table 1 provides a summary of how each response variable changed with a 4°C increase in temperature.

**Table 1:** Direction of change over a 4˚C increase in water temperature for each measured variable for all six species.

Variable		*Brycon amazonicus*	*Colossoma macropomum*	*Oreochromis niloticus*	*Labeo victorianus*	*Barbonymus gonionotus*	*Labeo pierrei*
Post-Acclimation	Survival	−	−	NS	NS	NS	NS
	SGR	NS	−	+	NS	NS	=
	W-at-L	+	NS	NS	NS	+	NS
	CTmax	+	+	+	+	+	+
	RMR	NS	NS	+	+	+	+
	MMR	=	NS	+	NS	+	NS
	AS	NS	NS	+	NS	NS	NS
	FAS	NS	NS	=	−	NS	−
Acute exposure	RMR	+	+	+	NS	+	NA
	MMR	=	+	NS	NS	NS	NA
	AS	NS	NS	NS	NS	NS	NA
	FAS	−	−	NS	NS	NS	NA

Plus sign = significant positive change. Minus sign = significant negative change. Equal sign = no change between Control and Control + 4°C, but significant overall effect of water temperature. NS = no significant effect of water temperature on variable. NA = Not available. SGR = specific growth rate. W-at-L = weight-at-length. CTmax = critical thermal maximum. RMR = Resting metabolic rate. MMR = Maximum metabolic rate. AS = Aerobic scope. FAS = Factorial aerobic scope.

### Survival, growth and weight-at-length

Fish survival was high at all temperatures over the course of the 21-d thermal acclimation for five out of six fish species (>87% survival, Table 2). Survival did not differ significantly among temperature treatments for the fish species from Uganda and Cambodia (Table 2). However, *Brycon amazonicus* survival at the control temperature was considerably lower (58%) than for other species, and decreased to 22% with + 4°C acclimation. Survival of the other Brazil species, *C. macropomum*, was also inversely related to treatment temperature, but survival was much higher than for *Brycon amazonicus*.

**Table 2:** Survival (%), specific growth rate (SGR; % per day) and weight-at-length (g) following thermal acclimation (21 d).

Country	Species	Parameter	Statistical Output	Control	Control + 2°C	Control + 4°C
Brazil	*Brycon amazonicus*	Survival (N)	*F*= 6.261; df = 2,6; *P* = 0.034	58 ± 7^a^ (48)	48 ± 7^ab^ (48)	22 ± 7^b^ (44)
		SGR (N)	*T*: *F* = 2.022; df = 2,6.922; *P* = 0.203 R: *F*= 1.991; df = 5,17; *P* = 0.132	4.504 ± 0.252 (13)	4.341 ± 0.259 (10)	2.859 ± 0.573 (2)
		Weight-at-length SL = 12.29 (N)	*T*: *F* = 10.287; df = 2,7.797; *P* = 0.007 R: *F* = 1.528; df = 6,18; *P* = 0.225 C: *F* = 197.965; df = 1,18; *P* < 0.001	34.91^a^ [32.06–37.93] (12)	35.16^a^ [31.92–38.82] (10)	49.32^b^ [43.85–55.59] (6)
	*Colossoma macropomum*	Survival	*F*= 9.000; df = 2,6; *P* = 0.016	100 ± 2^a^ (45)	93 ± 2^ab^ (42)	87 ± 2^b^ (39)
		SGR (N)	*T*: *F* = 19.103; df = 2,6.151; *P* = 0.002 R: *F*= 1.687; df = 6,55; *P* = 0.142	2.661 ± 0.085^a^ (24)	2.773 ± 0.093^a^ (21)	1.791 ± 0.097^b^ (19)
		Weight-at-length SL = 12.52 (N)	T: *F*= 1.224; df = 2,6.259; *P* = 0.356 R: *F* = 1.654; df = 6,62; *P* = 0.148 C: *F* = 878.391; df = 1,62; *P* < 0.001	58.48 [56.88–59.98] (24)	56.75 [55.21–58.34] (24)	56.36 [54.83–57.94] (24)
Uganda	*Oreochromis niloticus*	Survival (N)	*F*= 0.600; df = 2,6; *P* = 0.579	96 ± 6 (45)	87 ± 6 (45)	91 ± 6 (45)
		SGR (N)	T: *F* = 37.315; df = 2,6.379; *P* < 0.0001 R: *F* = 0.151; df = 6, 59; *P* = 0.988	−0.22 ± 0.057^a^ (23)	0.034 ± 0.055^b^ (24)	-0.009 ± 0.060^b^ (21)
		Weight-at-length SL = 9.38 (N)	T: *F*= 2.779; df = 2,5.992; *P* = 0.481 R: *F*= 0.366; df = 6,62; *P* = 0.034 C: *F* = 455.052; df = 1,62; *P* = 0.880	28.38 [27.48–29.31] (24)	28.12 [27.23–29.04] (24)	29.04 [28.12–29.92] (24)
	*Labeo victorianus*	Survival (N)	*F*= 0.892; df = 2,6; *P* = 0.458	92 ± 5 (36)	100 ± 5 (38)	90 ± 5 (39)
		SGR (N)	T: *F* = 0.353; df = 2,6.036; *P* = 0.716 R: *F*= 1.463; df = 6, 57; *P* = 0.207	−0.435 ± 0.036 (20)	−0.484 ± 0.033 (24)	−0.453 ± 0.035 (22)
		Weight-at-length SL = 11.41 (N)	T: *F*= 0.753; df = 2,5.902; *P* = 0.203 R: *F*= 1.508; df = 6,60; *P* = 0.131 C: *F* = 2042.919; df = 1,60; *P* = 0.971	21.73 [21.04–22.49] (22)	21.73 [21.04–22.44] (24)	21.09 [20.42–21.78] (24)
Cambodia	*Barbonymus gonionotus*	Survival (N)	*F*= 1.000; df = 2,6; *P* = 0.422	100 ± 1 (45)	100 ± 1 (45)	98 ± 1 (45)
		SGR (N)	T: *F* = 0.819; df = 2,6.009; *P* = 0.485 R: *F*= 2.216; df = 6, 61; *P* = 0.053	0.248 ± 0.033 (23)	0.312 ± 0.032 (24)	0.229 ± 0.033 (23)
		Weight-at-length SL = 9.88 (N)	T: *F*= 6.720; df = 2,5.936; *P* = 0.030 R: *F*= 1.191; df = 6,62; *P* = 0.323 C: *F*= 129.400; df = 1,62; *P* < 0.001	29.04^a^ [27.86–30.20] (24)	31.62^b^ [30.41–32.88] (24)	32.21^b^ [30.97–33.50] (24)
	*Labeo pierrei*	Survival (N)	*F* = 0.000; df = 2,6; *P* = 1.000	100 ± 2 (45)	100 ± 2 (45)	100 ± 2 (45)
		SGR (N)	T: *F* = 3.041; df = 2,4.971; *P* = 0.137 R: *F*= 1.365; df = 5, 54; *P* = 0.257	−0.089 ± 0.020^ab^ (22)	−0.030 ± 0.018^a^ (16)	−0.101 ± 0.015^b^ (24)
		Weight-at-length SL = 9.48 (N)	T: *F* = 2.586; df = 2,4.551; *P* = 0.532 R: *F*= 0.975; df = 5,55; *P* = 0.081 C: *F* = 330.116; df = 1,55; *P* = 0.857	20.09 [19.68–20.56] (24)	19.95 [19.41–20.46] (16)	19.45 [19.01–19.86] (24)

The effect of temperature treatment on survival and SGR was tested using a one-way ANOVA with ‘replicates’ nested within treatment as a factor to control for any potential treatment tank effect. Survival and specific growth rates are shown as means ± SEM. Differences in fish body size between temperature treatments post-acclimation were examined using an ANCOVA of log_10_ fish weight by treatment with log_10_ fish standard length as the covariate, with ‘replicates’ nested as above. When the interaction between temperature treatment and log_10_ fish standard length was not significant, it was removed from the model. Weight-at-length is presented as means [lower—upper 95% confidence interval]. For a given species, values with different letters differ significantly (Holm–Sidak); *P* ≤ 0.05. In the absence of a significant difference between temperature treatments, letters were omitted. *N*: number of individuals. SL: standard length (cm). df: degrees of freedom. *T*: Temperature treatment. R: Replicate. C: Covariate (log_10_SL). Control temperature in Brazil and Cambodia was 31°C whereas in Uganda Control temperature was 27°C.

Specific growth rate (SGR) during the treatment period varied widely among the various species. *Brycon amazonicus* (Brazil), *C. macropomum* (Brazil) and *Barbonymus gonionotus* (Cambodia) had positive growth rates at all three treatment temperatures (Table 2). In contrast, SGR was negative for *L. victorianus* (Uganda) and *L. pierrei* (Cambodia) in all temperature treatments (Table 2). SGR also differed within some species in response to temperature. In *C. macropomum*, SGR was lower at control +4°C than at the other temperatures, whereas in *L. pierrei*, SGR at control +4°C was lower than at control +2°C (Table 2). Conversely, SGR was greater at control +2°C and control +4°C compared to control in *O. niloticus* (Table 2). SGR did not differ between the temperature treatments for the other three species (*Brycon amazonicus, L. victorianus, Barbonymus gonionotus*; Table 2).

Fish weight-at-length at the end of the thermal acclimation period for *Brycon amazonicus* was higher in the control +4°C treatment than in either of the other two temperatures (Table 2), despite the fact that only 22% of the fish survived, suggesting some selection based on fish condition. Weight-at-length of *Barbonymus gonionotus* was higher in the control +2°C and control +4°C groups compared to the control treatment (Table 2). Fish weight-at-length did not change with treatment temperature in any of the other four species.

### Oxygen uptake

**Figure 2:**
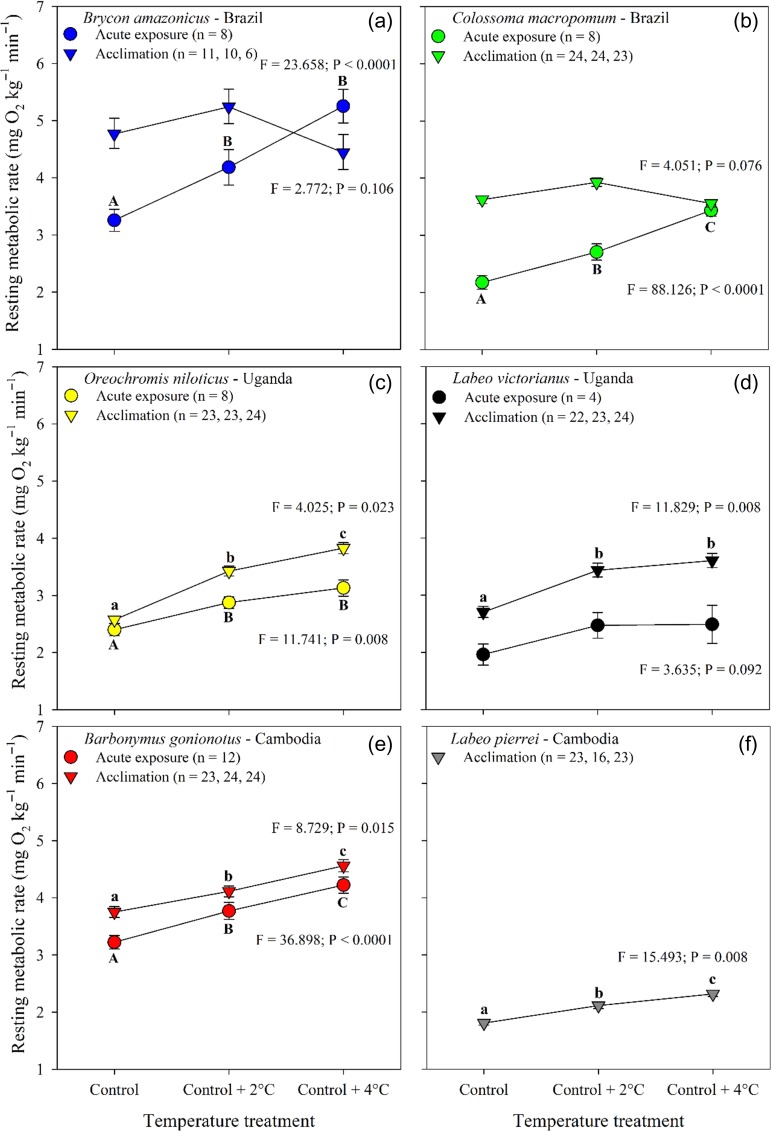
Resting metabolic rate (RMR) for six tropical freshwater fishes measured during an acute exposure (16 h; circles) and following thermal acclimation (21 d; triangles) to three water temperatures. Data are shown as model estimates of the mean ± SEM. The effect of temperature on RMR during the acute exposure was examined using a one-way repeated measures ANOVA that included temperature sequence group as a between-subject factor to control for the effect of treatment order on the responses, and its interaction with temperature treatment. For the post-acclimation data, temperature treatment effect on log_10_ total RMR was assessed using an ANCOVA with log_10_ fish mass as a covariate. ANCOVA included ‘replicates’ nested within treatment to account for tank effect. For a given species, means with different letters differ significantly from each other within each exposure period (Holm–Sidak; *P* ≤ 0.05; Supplementary Tables S1 and S2). Upper case letters = acute exposure whereas lower case letters = post-acclimation.

RMR measured in our acute and post-acclimation trials ranged between 2 and 5 mg O_2_ kg^−1^ min^−1^ (Fig. 2). An increase in temperature was generally associated with an increase in RMR, although other patterns were also observed (Fig. 2). An acute increase in temperature increased RMR over control conditions for four species but not for *L. victorianus*, where RMR was independent of acute increases in temperature (Fig. 2, Supplementary Table S1; *L. pierrei* was omitted from all acute temperature analyses, see methods). Following the 21-d acclimation, a +2°C increase in water temperature increased RMR compared with the control temperature in all species except for *Brycon amazonicus* and *C. macropomum* where RMR did not vary among temperature treatments (Fig. 2, Supplementary Table S2). Acclimation to +4°C further increased RMR in *O. niloticus*, *Barbonymus gonionotus* and *L. pierrei*, whereas it remained similar to RMR at control +2°C in *L. victorianus* (Fig. 2, Supplementary Table S2). A qualitative comparison of results for the acute warming and post-acclimation experiments shows that RMR values following the 21-d acclimation to the control treatment were higher than at comparable temperatures during acute exposure in *C. macropomum*, *L. victorianus*, and *Barbonymus gonionotus*, and for control and control +2 in the two Amazonian species (Fig. 2b,d,e).

**Figure 3:**
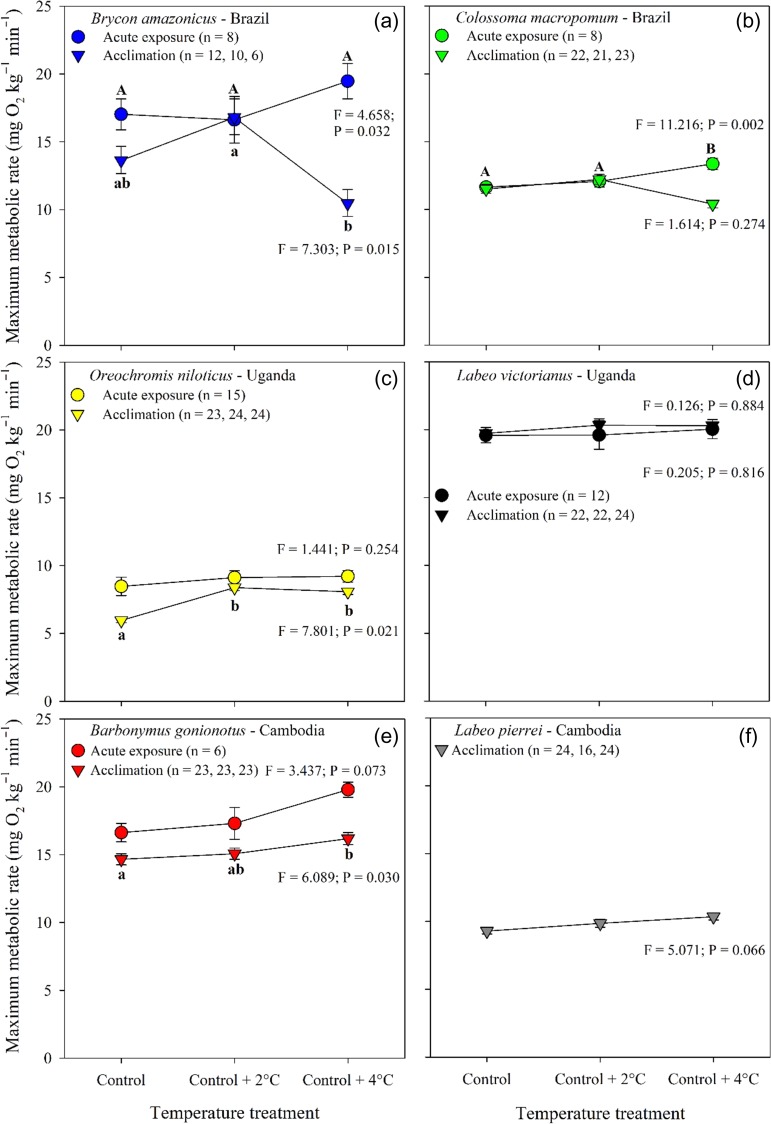
Maximum metabolic rate (MMR) for six tropical freshwater fishes measured during an acute exposure (16 h; circles) and following thermal acclimation (21 d; triangles) to three water temperatures. Data are shown as model estimates of the mean ± SEM. The effect of temperature on MMR during the acute exposure was examined using a one-way repeated measures ANOVA that included temperature sequence group as a between-subject factor to control for the effect of treatment order on the responses, and its interaction with temperature treatment. For the post-acclimation data, temperature treatment effect on log_10_ total MMR was assessed using an ANCOVA with log_10_ fish mass as a covariate. ANCOVA included ‘replicates’ nested within treatment to account for tank effect. For a given species, means with different letters differ significantly from each other within each exposure period (Holm–Sidak; *P* ≤ 0.05; Supplementary Tables S1 and S4). Upper case letters = acute exposure whereas lower case letters = post-acclimation.

MMR values ranged between 5 mg O_2_ kg^−1^ min^−1^ and 20 mg O_2_ kg^−1^ min^−1^ (Fig 3). In the acute exposure experiment MMR increased between the control and control +4 only in *C. macropomum* (Fig. 3b, Supplementary Table S1), whereas MMR was unchanged in *O. niloticus*, *L. victorianus*, and *Barbonymus gonionotus* (Fig. 3c,d,e, Supplementary Table S1). While temperature treatment affected MMR in *Brycon amazonicus*, the pairwise comparisons did not show differences between treatments (Fig. 3a, Supplementary Table S1). Post-acclimation MMR increased significantly in *O. niloticus* and *Barbonymus gonionotus* (Fig. 3c,e, Supplementary Table S3), and was unchanged in *C. macropomum, L. victorianus*, and *L. peirrei* (Fig. 3b,d,f). In *Brycon amazonicus*, MMR decreased between control +2°C and control +4°C, but neither differed from that of the control group (Fig. 3a, Supplementary Table S3). A qualitative comparison of the two experiments suggests a lower MMR in response to 21 d acclimation in *O. niloticus and Barbonymus gonionotus*. The pattern was less clear in *Brycon amazonicus*.

**Figure 4:**
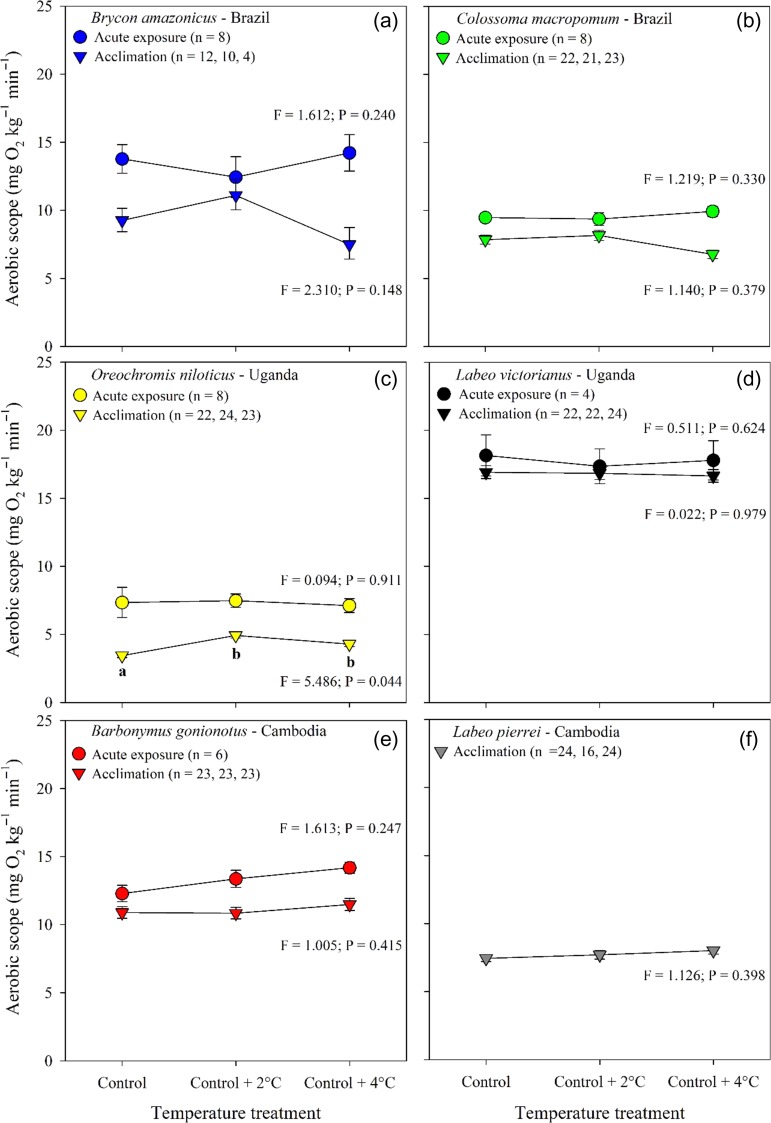
Aerobic scope (AS = MMR–RMR) for six tropical freshwater fishes measured during an acute exposure (16 h; circles) and following thermal acclimation (21 d; triangles) to three water temperatures. Data are shown as model estimates of the mean ± SEM. The effect of temperature on AS during the acute exposure was examined using a one-way repeated measures ANOVA that included temperature sequence group as a between-subject factor to control for the effect of treatment order on the responses, and its interaction with temperature treatment. For the post-acclimation data, temperature treatment effect on log_10_ total AS was assessed using an ANCOVA with log_10_ fish mass as a covariate. ANCOVA included ‘replicates’ nested within treatment to account for tank effect. For a given species, means with different letters differ significantly from each other within each exposure period (Holm–Sidak; *P* ≤ 0.05; Supplementary Tables S1 and S5). Upper case letters = acute exposure whereas lower case letters = post-acclimation.

Both acute exposure and thermal acclimation had only modest effects on AS. AS was maintained over a 4˚C acute increase in water temperature in the five species (*Brycon amazonicus, C. macropomum, O. niloticus, L. victorianus, Barbonymus gonionotus*, Fig. 4a,b,c,d,e, Supplementary Table S1). In the 21 d acclimation, five species maintained AS across the three experimental temperatures, while *O. niloticus* showed an increase in AS (Fig. 4, Supplementary Table S4). In all six species, AS of acclimated fish tended to be lower than at the same temperatures during acute exposures (Fig. 4).

**Figure 5:**
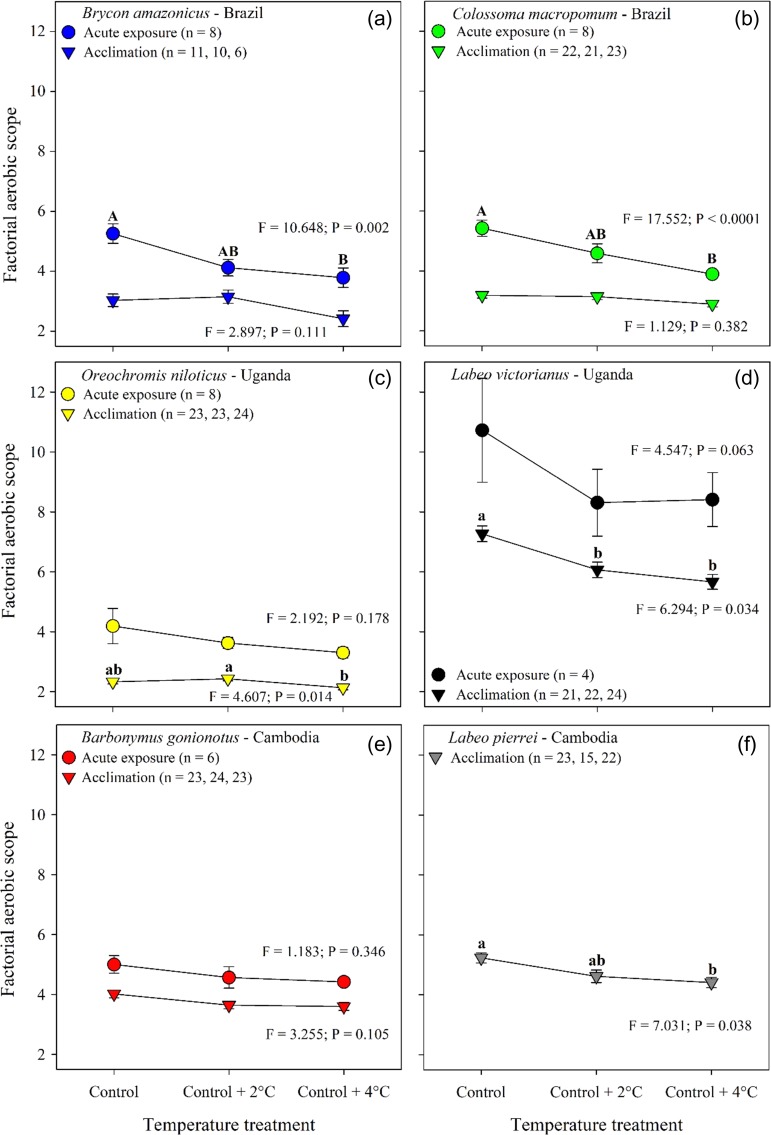
Factorial aerobic scope (FAS = MMR–RMR^−1^) for six tropical freshwater fishes measured during an acute exposure (16 h; circles) and following thermal acclimation (21 d; triangles) to three water temperatures. Data are shown as model estimates of the mean ± SEM. The effect of temperature on FAS during the acute exposure was examined using a one-way repeated measures ANOVA that included temperature sequence group as a between-subject factor to control for the effect of treatment order on the responses, and its interaction with temperature treatment. For the post-acclimation data, temperature treatment effect on log_10_ FAS was assessed using an ANCOVA with log_10_ fish mass as a covariate. ANCOVA included ‘replicates’ nested within treatment to account for tank effect. For a given species, means with different letters differ significantly from each other within each exposure period (Holm–Sidak; *P* ≤ 0.05; Supplementary Tables S1 and S6). Upper case letters = acute exposure whereas lower case letters = post-acclimation.

FAS did not change significantly with a 4˚C acute increase in water temperature for three species (Fig. 5c,d,e, Supplementary Table S1), but decreased with increasing water temperature in *Brycon amazonicus* and *C. macropomum* (Fig. 5a,b, Supplementary Table S1). Post-acclimation, FAS remained unchanged in *Brycon amazonicus*, *C. macropomum* and *Barbonymus gonionotus* (Fig. 5a,b,e, Supplementary Table S5), whereas FAS decreased with increasing temperature in the two *Labeo* species (Fig. 5d,f, Supplementary Table S5). In *O. niloticus*, FAS post-acclimation differed significantly between control +2°C and control +4°C, but neither was significantly different from FAS measured at the control water temperature (Fig. 5c, Supplementary Table S5).

### Critical thermal maximum

**Figure 6:**
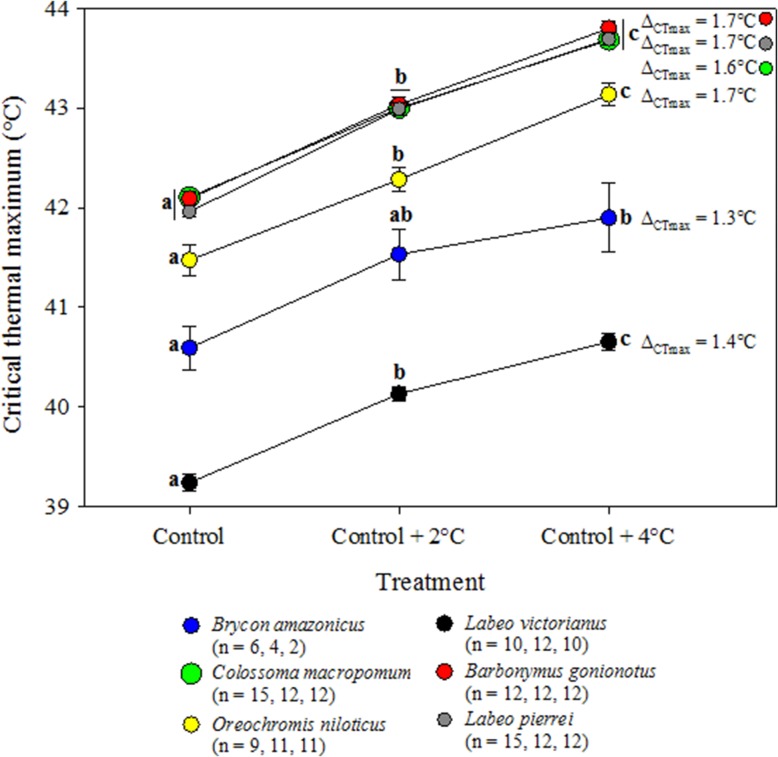
Critical thermal maximum compared between temperature treatments. Data are shown as means ± SEM. For a given species, symbols with different letters differ significantly (Holm–Sidak; *P* ≤ 0.05; Supplementary Table S7).

All six species showed significant increases in CTmax with an increase in treatment temperature, although a numerical increase in CTmax of *Brycon amazonicus* between control +2°C and control +4°C temperatures did not reach statistical significance (Fig. 6, Supplementary Table S6). Depending on the species, the gain in CTmax ranged from 1.3 to 1.7°C for a 4°C increase in temperature, with the highest values observed in the two Cambodia species (*Barbonymus gonionotus* and *L. pierrei*) as well as *C. macropomum* (Fig. 6).

## Discussion

In their comprehensive review of climate change impact studies on inland fishes, Myers *et al.* (2017) noted studies of the fishes of ‘Asia, Africa and South America are notably underrepresented…’ and that ‘The lack of research that predicts how climate change will affect these vital natural resources in developing countries is a substantial knowledge gap in climate change and fisheries research, and could have major detrimental consequences for people in these regions reliant on inland fishes.’ Here, we quantified the responses of six equatorial inland fish species to acute exposure and 21 d of acclimation to realistic projections of water temperature increases (i.e. +2°C and +4°C). We present our results as evaluations of the applicability of two hypotheses: that equatorial fishes will lack capacity for adaptation and will be severely challenged by warming water temperatures (CVH), and that a decline in AS will be the causal mechanism of the whole-animal performance decline (OCLTT). Support for the first hypothesis would likely translate to negative consequences for biodiversity and food security of dependent human communities, while support for the second would help to establish a mechanistic understanding of how climate warming may impact tropical ectotherms.

In contrast to the prediction of widespread sensitivity to warming of tropical ectotherms postulated in the CVH, the six equatorial species we studied provided some evidence of an ability to tolerate and perform at temperatures up to 4°C above their current summer maxima. For example, five of the six species had survival rates >87% and four had no change in SGR over the 21 d treatment period. Additionally, compensatory thermal acclimation was seen definitively in the five species with good survival as an increase in their upper thermal tolerance (e.g. CTmax), with the sixth species (*Brycon amazonicus*) approaching statistical significance. This thermal compensation can be expressed as a change in CTmax per °C of increase in acclimation temperature, for which *L. pierrei* showed the greatest (ratio = 0.43) improvement in thermal tolerance and *Brycon amazonicus* the lowest (ratio = 0.33). While these ratios illustrate that there is not complete compensation for the temperature increase, they are in line with ratios reported for fish species from an extremely wide geographic range: the Arctic (*Boreogadus saida* = 0.43, Drost *et al.* (2016)), the Antarctic (*Pagothenia borchgrevinki* = 0.55, *Trematomus pennelii* = 0.53, *T. hansoni* = 0.39, *T. bernacchii* = 0.24, *Lycodichthys dearborni* = 0.33, *Pachycara brachycephalum* = 0.44, *Gobionotothen gibberifrons* = 0.3, *Notothenia coriiceps* = 0.21; Bilyk and DeVries, 2011), temperate regions (range from 0.27 to 0.50 with a mean ± SD of 0.41 ± 0.07°C for 20 North American freshwater species (Beitinger *et al.*, 2000)) and the tropics (*Horabagrus brachysoma* = 0.26, Dalvi *et al.* (2009); *Albula vulpes* = 0.52, Murchie *et al.* (2011); *Pseudocrenilabrus multicolour victoriae* = 0.43, McDonnell and Chapman (2015); *Paracheirodon simulans =* 0.50, Campos *et al.* (2017)). The low thermal compensation displayed by *Brycon amazonicus* is similar to that reported for another amazonian fish, *Paracheirodon axelrdi* (Campos *et al.* 2017), and combined with its very poor survival at the highest acclimation temperature suggests a greater susceptibility to global warming compared with the other species examined in this study and should be further explored.

Measurements of AS, which has been hypothesized to be a key predictor of a fish’s response to warming waters (Pörtner, 2002; Pörtner and Farrell, 2008; Pörtner and Knust, 2007), likewise provide evidence of tolerance to warming as each of the fish species studied here maintained or slightly increased AS with warming temperatures in both acute and acclimation trials. Numerous other tropical fishes have previously been shown to maintain AS with warming to temperatures close to their upper thermal limits, for example, *Lates calcarifer* acclimated to temperatures up to 38°C (a temperature near CTmax) for 5 weeks (Norin *et al.*, 2014), *L. niloticus* acclimated to temperatures up to 31.5°C (ambient +4°C) for an average of 5 days (Chrétien and Chapman, 2016), *L. niloticus* acclimated to temperatures up to 31°C for 3 weeks (ambient +4°C) (Nyboer and Chapman, 2013), and *Acanthochromis polyacanthus* and *Pomacentrus moluccensis* following up to 14 days of acclimation to 33°C and 34°C, respectively (Rummer *et al.*, 2013). Tropical fishes have also been previously shown to have a lower AS after warm acclimation when compared with acute warming, e.g. Nile perch (*Lates niloticus*) acclimated for 21 days to ambient temperature and +4°C (Nyboer and Chapman (2013)) and barramundi (*Lates calcarifer)* after 5 weeks of acclimation to 38°C (Norin *et al.*, 2014). In the present study, there was a tendency for AS of the acclimated fish to be lower than the acutely warmed fish in part because MMR was maintained after warm acclimation in 5 of the species and significantly increased in the other species, whereas RMR increased in 4 species and was maintained in the other two species. The two Cambodia and the two Uganda species had similar survival after warm acclimation, and in three of these four species the fish experienced a negative SGR under control conditions. Given these results and the poor survival of the *Brycon* (see below), a rigorous assessment of OCLTT predictions (e.g. relationships between survival, SGR and AS following warm acclimation) was possible only for one species, *Colossoma macropomum*. For *Colossoma macropomum* neither RMR, MMR nor AS changed significantly with warm acclimation despite the fall off in SGR and survival, a result that does not lend support for the OCLTT. Furthermore, while survival was generally high across all species, the two Brazil species suffered decreased survival after acclimation to +4°C, and in the case of *Brycon amazonicus* this was on top of the 42% mortality of fish at the control temperature. The mechanism causing mortality in *Brycon amazonicus* was not investigated beyond the measurements included here, but it seems likely that elevated temperatures exacerbated the species’ known tendency for aggressive behaviours and cannibalism (Wolkers *et al.*, 2014), as suggested by the surviving fish at the highest temperature having an elevated weight-at-length. A similar temperature-aggression relationship was also noted for the African cichlid *Pseudocrenilabrus multicolor victoriae* (L. Chapman, unpublished data).

Given the variable species-specific responses to acute warming and temperature acclimation, we suggest that future assessments will need to be species-specific and placed into the local context, especially given the increasing evidence for local adaptations of thermal performance within salmonid species (Chen *et al.*, 2013; Eliason *et al.*, 2011, 2013; Verhille *et al.*, 2016). In fact, we strongly caution against the broad generalizations of tropical ectotherm sensitivity to warming proposed by the CVH. Given that our CTmax data match up well with fishes from a broad geographic range including the temperate zone, it could be that tropical freshwater fishes are more like other fishes of the world and less like other tropical ectotherms. Furthermore, we suggest that while the OCLTT may remain a useful framework for the study of fish performance in simulated warming scenarios, it must be recognized that the response of AS to thermal acclimation varies widely among fish species (Farrell *et al.*, 2009; Fry, 1947; Jutfelt *et al.*, 2018). As such, we suggest AS should be viewed as just one among a suite of relevant performance measures that should be used in vulnerability assessments (Clark *et al.*, 2013), especially until we have a better appreciation of how and when a fish might exploit this maximum capacity (Farrell, 2016).

An overarching concern with our experimental design is that the comparison between acute and acclimation responses is confounded by the control temperature being defined as the current average maximum temperature experienced in each location (i.e. average water temperature measured during the warmest time of the day during the warmest months of the year). Thus, the fish were not necessarily experiencing this control temperature when they were either caught or purchased for the laboratory experiments. This was an inherent limitation of fish acquisition in these areas. Furthermore, the broad-scale applicability of our results as a first order vulnerability assessment of tropical inland fishes to climate warming is dependent upon the accuracy of our selected experimental water temperatures. While empirical records of water temperature data were available for the Brazilian habitats of the fish species that we used, empirical habitat data were limited for the Cambodia and Uganda species and we therefore relied to a greater extent on the professional judgement of our in-country collaborators. During the time course of our experiment, an independent study deployed numerous water temperature loggers into Tonle Sap Lake (Cambodia), part of the native range of both of our Cambodia fish species. While much of the measured water temperatures match up well with our selected conditions, there were a modest number of occasions where water temperatures in shallow littoral areas reached 38–40°C (G. Holtgrieve, U. Washington, personal communication), temperatures well above our ambient +4°C test treatment (35°C). We do not know the spatial extent of these warmer waters, whether our species were present in these locations, nor the behavioural response of these fishes if they encountered these elevated temperatures. Nevertheless, if wild populations of our species are currently experiencing water with temperatures that occasionally reach 38–40°C, then our results may not accurately reflect the true magnitude of adaptive capacity nor the true magnitude of threat of climate warming to tropical inland fishes. Certainly, questions regarding the interaction between spatial patterns in temperature and behaviours of freshwater tropical fishes is an area in need of additional study.

Despite these limitations, the present study on six species from three continents did reveal a consistent result of some concern for a future warming scenario. RMR and standard metabolic rate (SMR) are expected to steadily increase with warming as the energetic cost of maintenance increases due to increased rates of chemical reaction rates, which results in an increase in oxygen demand at the level of the mitochondria (McNab, 2002). Successful thermal acclimation, in the absence of other deleterious responses such as poor survival or weight loss, would be evidenced by a lower (compensated) RMR at a specific temperature relative to acute exposure at the same temperature, and perhaps an attenuated increase in RMR or SMR with further increases in water temperature (Schulte, 2015). Interestingly, we found that RMR increased after warm acclimation in five of the six species examined, suggesting that a 21-d exposure to ecologically relevant projections for increases in water temperature did not result in significant metabolic compensation. Moreover, RMR in acclimated fish was numerically higher than RMR after acute warming to the same temperature in some of the species (although it was not possible to test this statistically). This response may be a concern because for a fish to maintain body mass or grow at the same rate with a higher RMR, the fish must correspondingly increase food intake along with the associated risks and costs of increased foraging. Similar results showing no thermal compensation of RMR have been reported for three equatorial coral reef fishes (*Pomacentrus moluccensis*, *Dascyllus melanurus*, and *Chromis atripectoralis*) after 12–14 days of acclimation (Rummer *et al.*, 2013) and an African cichlid (*Pseudocrenilabrus multicolor victoriae*) after 3 days of acclimation (McDonnell and Chapman, 2015). All the same, the difference in RMR response of the Brazil species compared to the other species, as well as their poorer survival and reduced SGR with warm acclimation despite the increase in CTmax, highlights the species-specific differences among fishes and the need for more, and more realistic, studies with tropical freshwater species that are important to human food security via wild capture fisheries or aquaculture. For example, our experiments provided the fish with ample food and high, constant water quality. Overt disease, temperature fluctuations and inter-specific interactions were absent. These conditions obviously overlook many of the natural perturbations experienced by fish in the wild. Our ability to predict and plan for the future of critically important tropical inland fisheries and biodiversity conservation would benefit greatly from incorporating additional scenarios besides static warming under optimal holding conditions, such as fluctuating temperatures, variable food quantity and quality, and inter-specific interactions. However, we caution against interpreting our results as a ‘best-case’ scenario of the adaptive abilities of the tested species, as we did introduce certain externalities such as the stress of capture, transportation, handling and prolonged captivity. Also, greatly needed are studies that link fish behaviour to environmental conditions (e.g. using thermal loggers implanted in fish) to be sure of the realistic temperature exposures and their durations. Other aspects of climate change, such as how changing temperatures and wind speeds will affect the productivity of lake systems via altered stratification and nutrient availability, and the increasing frequency of extreme climatic events (Ficke *et al.*, 2007), will also need to be incorporated into data sets such as this one before a comprehensive evaluation of how tropical freshwater fish populations and inland fisheries will respond to climate change.

In the face of global climate warming, the persistence of tropical inland fishes will depend on their capacity to quickly respond to changes via acclimation and plasticity, or more gradually via genetic adaptation to temperature extremes that they have rarely if ever encountered. Their other option, to shift their distributions to more favourable environments, could compromise fisheries and threaten food security and livelihoods wherever inland fisheries make significant contributions to human well-being (Cooke *et al.*, 2016; Welcomme *et al.*, 2010) while simultaneously having dramatic effects on aquatic biodiversity and community structure (Grigaltchik *et al.*, 2012; Russell *et al.*, 2012). Of course, such range shifts may not even be possible as obligate lake dwellers will be constrained to their home waters and riverine fishes endemic to low gradient river segments may not be able to tolerate higher gradients common to higher elevation segments. The expansive modifications of freshwater systems and loss of system connectivity (e.g. via dams) further reduce options for range shifts. Although our results provide some hope that tropical freshwater fishes, and therefore the fisheries based upon them, will persist in the face of rising temperatures over the following decades, our results also illustrate the importance of attempting to limit the magnitude of temperature rise. Tried and true management focused on limiting rising water temperatures, such as limiting water extractions, protecting riparian zone vegetation in headwaters, implementing ecologically defensible environmental flow requirements for regulated rivers, and the protection and conservation of both genetic and phenotypic diversity (Myers *et al.*, 2017) should remain management priorities, as should the global need to drastically reduce greenhouse gas emissions.

## Supplementary Material

Supplementary Data
