# Supplementary material for: Predicted impacts of climate warming on aerobic performance and upper thermal tolerance of six tropical freshwater fishes spanning three continents
Source: Conserv Physiol. 2018 Oct 15;6(1):coy056. doi: 10.1093/conphys/coy056 (PMC6188536; doi:10.1093/conphys/coy056)
Supplement: Supplementary Data [file coy056_supplementary_table.docx]

**Table S1.** Statistical output for resting metabolic rate (RMR), maximum metabolic rate (MMR), aerobic scope (AS), and factorial aerobic scope (FAS) following acute exposure. The effect of temperature on MR was examined using a one-way repeated measures ANOVA. For *Brycon amazonicus* and *Colossoma macropomum*, the model included temperature sequence group as a between-subject factor to control for the effect of treatment order on the responses, and its interaction with temperature treatment. For the other three species, only one group was included in the statistical analysis. df = degrees of freedom.

| **Variable** | **Species** | **F** | **df (factor, error)** | **P** |
| --- | --- | --- | --- | --- |
| RMR | *Brycon amazonicus* | 23.658 | 2, 12 | <0.0001 |
|  | *Colossoma macropomum* | 88.126 | 2, 12 | <0.0001 |
|  | *Oreochromis niloticus* | 11.741 | 1.14, 14 | 0.008 |
|  | *Labeo victorianus* | 3.635 | 2, 6 | 0.092 |
|  | *Barbonymus gonionotus* | 36.898 | 2, 22 | <0.0001 |
| MMR | *Brycon amazonicus* | 4.658 | 2, 12 | 0.032 |
|  | *Colossoma macropomum* | 11.216 | 2, 12 | 0.002 |
|  | *Oreochromis niloticus* | 1.441 | 2, 28 | 0.254 |
|  | *Labeo victorianus* | 0.205 | 2, 22 | 0.816 |
|  | *Barbonymus gonionotus* | 3.437 | 2, 10 | 0.073 |
| AS | *Brycon amazonicus* | 1.612 | 2, 12 | 0.240 |
|  | *Colossoma macropomum* | 1.219 | 2, 12 | 0.330 |
|  | *Oreochromis niloticus* | 0.094 | 2, 14 | 0.911 |
|  | *Labeo victorianus* | 0.511 | 2, 6 | 0.624 |
|  | *Barbonymus gonionotus* | 1.613 | 2, 10 | 0.247 |
| FAS | *Brycon amazonicus* | 10.648 | 2, 12 | 0.002 |
|  | *Colossoma macropomum* | 17.552 | 2, 12 | < 0.0001 |
|  | *Oreochromis niloticus* | 2.192 | 1.136, 7.951 | 0.178 |
|  | *Labeo victorianus* | 4.547 | 2, 6 | 0.063 |
|  | *Barbonymus gonionotus* | 1.183 | 2, 10 | 0.346 |

**Table S2.** Statistical output for resting metabolic rate (RMR) post-acclimation. Temperature treatment effects on log_10_ total RMR were assessed using an ANCOVA with log_10_ fish mass as a covariate. Each ANCOVA included “replicates” nested within treatment to account for tank effect. df = degrees of freedom.

| **Species** | **Source** | **F** | **df (factor, error)** | **P** |
| --- | --- | --- | --- | --- |
| *Brycon amazonicus* | Treatment | 2.772 | 2, 10.939 | 0.106 |
|  | Replicate | 0.555 | 6, 17 | 0.760 |
|  | logMass | 101.150 | 1, 17 | <0.0001 |
| *Colossoma macropomum* | Treatment | 4.051 | 2, 6.132 | 0.076 |
|  | Replicate | 1.851 | 6, 61 | 0.104 |
|  | logMass | 384.609 | 1, 61 | <0.0001 |
| *Oreochromis niloticus* | Treatment | 4.025 | 2, 59.528 | 0.023 |
|  | Replicate | 4.515 | 6, 58 | 0.001 |
|  | logMass | 150.649 | 1, 58 | <0.0001 |
|  | Treatment*logMass | 3.148 | 2, 58 | 0.050 |
| *Labeo victorianus* | Treatment | 11.829 | 2, 5.948 | 0.008 |
|  | Replicate | 1.534 | 6, 59 | 0.183 |
|  | logMass | 284.406 | 1, 59 | <0.0001 |
| *Barbonymus gonionotus* | Treatment | 8.729 | 2, 6.364 | 0.015 |
|  | Replicate | 1.863 | 6, 61 | 0.102 |
|  | logMass | 153.125 | 1, 61 | <0.0001 |
| *Labeo pierrei* | Treatment | 15.493 | 2, 4.854 | 0.008 |
|  | Replicate | 2.355 | 5, 53 | 0.053 |
|  | logMass | 92.962 | 1, 53 | <0.0001 |

**Table S3.** Statistical output for maximum metabolic rate (MMR) post-acclimation. Temperature treatment effects on log_10_ total MMR were assessed using an ANCOVA with log_10_ fish mass as a covariate. Each ANCOVA included “replicates” nested within treatment to account for tank effect. df = degrees of freedom.

| **Species** | **Source** | **F** | **df (factor, error)** | **P** |
| --- | --- | --- | --- | --- |
| *Brycon amazonicus* | Treatment | 7.303 | 2, 8.375 | 0.015 |
|  | Replicate | 1.047 | 6, 18 | 0.429 |
|  | logMass | 20.824 | 1, 18 | 0.536 |
| *Colossoma macropomum* | Treatment | 1.614 | 2, 6.111 | 0.274 |
|  | Replicate | 4.833 | 6, 56 | <0.0001 |
|  | logMass | 162.709 | 1, 56 | <0.0001 |
| *Oreochromis niloticus* | Treatment | 7.801 | 2, 5.993 | 0.021 |
|  | Replicate | 6.096 | 6, 61 | <0.0001 |
|  | logMass | 85.492 | 1, 61 | <0.0001 |
| *Labeo victorianus* | Treatment | 0.126 | 2, 5.979 | 0.884 |
|  | Replicate | 4.169 | 6, 58 | 0.002 |
|  | logMass | 533.634 | 1, 58 | <0.0001 |
| *Barbonymus gonionotus* | Treatment | 6.089 | 2, 6.883 | 0.030 |
|  | Replicate | 0.494 | 6, 59 | 0.811 |
|  | logMass | 39.280 | 1, 59 | <0.0001 |
| *Labeo pierrei* | Treatment | 5.071 | 2, 4.740 | 0.066 |
|  | Replicate | 0.934 | 5, 55 | 0.466 |
|  | logMass | 41.576 | 1, 55 | <0.0001 |

**Table S4.** Statistical output for aerobic scope (AS) post-acclimation. Temperature treatment effects on log_10_ total AS were assessed using an ANCOVA with log_10_ fish mass as a covariate. Each ANCOVA included “replicates” nested within treatment to account for tank effect. df = degrees of freedom.

| **Species** | **Source** | **F** | **df (factor, error)** | **P** |
| --- | --- | --- | --- | --- |
| *Brycon amazonicus* | Treatment | 2.310 | 2, 10.420 | 0.148 |
|  | Replicate | 0.941 | 6, 16 | 0.493 |
|  | logMass | 9.835 | 1, 16 | 0.006 |
| *Colossoma macropomum* | Treatment | 1.140 | 2, 6.118 | 0.379 |
|  | Replicate | 4.563 | 6, 56 | 0.001 |
|  | logMass | 78.124 | 1, 56 | <0.0001 |
| *Oreochromis niloticus* | Treatment | 5.486 | 2, 5.994 | 0.044 |
|  | Replicate | 3.240 | 6, 59 | 0.008 |
|  | logMass | 16.590 | 1, 59 | <0.0001 |
| *Labeo victorianus* | Treatment | 0.022 | 2, 5.977 | 0.979 |
|  | Replicate | 3.815 | 6, 58 | 0.003 |
|  | logMass | 365.290 | 1, 58 | <0.0001 |
| *Barbonymus gonionotus* | Treatment | 1.005 | 2, 6.695 | 0.415 |
|  | Replicate | 0.623 | 6, 59 | 0.711 |
|  | logMass | 13.282 | 1, 59 | 0.001 |
| *Labeo pierrei* | Treatment | 1.126 | 2, 4.759 | 0.398 |
|  | Replicate | 1.010 | 5, 55 | 0.421 |
|  | logMass | 19.573 | 1, 55 | <0.0001 |

**Table S5.** Statistical output for factorial aerobic scope (FAS) post-acclimation. Temperature treatment effects on log_10_ total FAS were assessed using an ANCOVA with log_10_ fish mass as a covariate. Each ANCOVA included “replicates” nested within treatment to account for tank effect. df = degrees of freedom.

| **Species** | **Source** | **F** | **df (factor, error)** | **P** |
| --- | --- | --- | --- | --- |
| *Brycon amazonicus* | Treatment | 2.897 | 2, 8.291 | 0.111 |
|  | Replicate | 0.863 | 6, 17 | 0.541 |
|  | logMass | 2.625 | 1, 17 | 0.124 |
| *Colossoma macropomum* | Treatment | 1.129 | 2, 6.240 | 0.382 |
|  | Replicate | 2.542 | 6, 56 | 0.030 |
|  | logMass | 0.130 | 1, 56 | 0.720 |
| *Oreochromis niloticus* | Treatment | 4.607 | 2, 58.708 | 0.014 |
|  | Replicate | 1.533 | 6, 58 | 0.184 |
|  | logMass | 18.719 | 1, 58 | <0.0001 |
|  | Treatment*logMass | 3.892 | 2, 58 | 0.026 |
| *Labeo victorianus* | Treatment | 6.294 | 2, 5.990 | 0.034 |
|  | Replicate | 1.649 | 6, 57 | 0.151 |
|  | logMass | 1.146 | 1, 57 | 0.289 |
| *Barbonymus gonionotus* | Treatment | 3.255 | 2, 6.442 | 0.105 |
|  | Replicate | 0.933 | 6, 60 | 0.478 |
|  | logMass | 7.311 | 1, 60 | 0.009 |
| *Labeo pierrei* | Treatment | 7.031 | 2, 4.725 | 0.038 |
|  | Replicate | 0.898 | 5, 51 | 0.490 |
|  | logMass | 1.494 | 1, 51 | 0.227 |

**Table S6.** Statistical output for critical thermal maximum (CTmax). A one-way ANOVA tested treatment temperature effects on CTmax, with ‘replicates’ nested within treatment to control for an effect of treatment tank. df = degrees of freedom.

| **Species** | **Source** | **F** | **df (factor, error)** | **P** |
| --- | --- | --- | --- | --- |
|  |  |  |  |  |
| *Brycon amazonicus* | Treatment | 8.391 | 2, 6.945 | 0.014 |
|  | Replicate | 0.76 | 5, 4 | 0.622 |
| *Colossoma macropomum* | Treatment | 54.123 | 2, 6.038 | <0.0001 |
|  | Replicate | 4.183 | 6, 30 | 0.004 |
| *Oreochromis niloticus* | Treatment | 19.029 | 2, 6.669 | 0.002 |
|  | Replicate | 2.085 | 6, 22 | 0.097 |
| *Labeo victorianus* | Treatment | 76.457 | 2, 6.372 | <0.0001 |
|  | Replicate | 1.022 | 6, 23 | 0.436 |
| *Barbonymus gonionotus* | Treatment | 536.085 | 2, 6 | <0.0001 |
|  | Replicate | 0.246 | 6, 27 | 0.957 |
| *Labeo pierrei* | Treatment | 212.303 | 2, 6.153 | <0.0001 |
|  | Replicate | 1.054 | 6, 30 | 0.411 |
